# X-ray Structure and Enzymatic Activity Profile of a Core Papain-like Protease of MERS Coronavirus with utility for structure-based drug design

**DOI:** 10.1038/srep40292

**Published:** 2017-01-12

**Authors:** Jozlyn R. Clasman, Yahira M. Báez-Santos, Robert C. Mettelman, Amornrat O’Brien, Susan C. Baker, Andrew D. Mesecar

**Affiliations:** 1 Department of Biological Sciences, Purdue University, West Lafayette, IN, USA; 2 Department of Microbiology and Immunology, Loyola University Chicago, Stritch School of Medicine, Maywood, IL, USA; 3 Department of Biochemistry, Purdue University, West Lafayette, IN, USA; 4 Center for Cancer Research, Purdue University, West Lafayette, IN, USA.

## Abstract

Ubiquitin-like domain 2 (Ubl2) is immediately adjacent to the N-terminus of the papain-like protease (PLpro) domain in coronavirus polyproteins, and it may play a critical role in protease regulation and stability as well as in viral infection. However, our recent cellular studies reveal that removing the Ubl2 domain from MERS PLpro has no effect on its ability to process the viral polyprotein or act as an interferon antagonist, which involves deubiquitinating and deISGylating cellular proteins. Here, we test the hypothesis that the Ubl2 domain is not required for the catalytic function of MERS PLpro *in vitro*. The X-ray structure of MERS PLpro-∆Ubl2 was determined to 1.9 Å and compared to PLpro containing the N-terminal Ubl2 domain. While the structures were nearly identical, the PLpro-∆Ubl2 enzyme revealed the intact structure of the substrate-binding loop. Moreover, PLpro-∆Ubl2 catalysis against different substrates and a purported inhibitor revealed no differences in catalytic efficiency, substrate specificity, and inhibition. Further, no changes in thermal stability were observed between enzymes. We conclude that the catalytic core of MERS PLpro, i.e. without the Ubl2 domain, is sufficient for catalysis and stability *in vitro* with utility to evaluate potential inhibitors as a platform for structure-based drug design.

Coronaviruses (CoVs) are enveloped, positive sense, single-stranded RNA viruses that cause mild to severe upper respiratory tract infections in humans. Approximately 10 years after emergence of the severe acute respiratory syndrome coronavirus (SARS-CoV) in 2002/2003, Middle East respiratory syndrome coronavirus (MERS-CoV) emerged and has been identified so far in 26 countries with a case-fatality rate over 30%^1^,^2^. Although these CoVs are well-recognized global pathogens, there are no antiviral interventions available. Thus, a better understanding of the molecular mechanisms that facilitate viral pathogenesis and replication may permit the design of targeted therapeutics against CoVs.

MERS-CoV is classified in the sub-lineage C genus *Betacoronavirus* with a conserved genomic size of ~30 kb among other CoVs^2^. The first 22 kilobases located at the 5′-end of the RNA genome is encoded in two open reading frames (ORF1a/ORF1b) that are translated by host ribosomes to generate two respective viral polyproteins (pp1a & pp1ab). Pp1a and pp1ab are processed by two virus-encoded cysteine proteases, termed the papain-like protease (PLpro) and the 3C-like protease (3CLpro). Together, these two proteases cleave the polyproteins to produce 16 nonstructural proteins (nsps), which are essential for the formation of the replicase complex and hence RNA replication. This study focuses on the multifunctional and putative drug target, PLpro, located in nonstructural protein 3 (nsp3; Fig. 1a).

In addition to its function of cleaving the viral polyprotein into the requisite nsps, SARS-CoV PLpro is also a viral ubiquitin-specific protease (vUSP), having a structural fold almost identical to the human USP family^3^,^4^,^5^. SARS PLpro is a highly efficient deubiquitinating (DUB) enzyme having the ability to rapidly hydrolyze isopeptide bonds of proteins that are post-translationally modified by cellular ubiquitin-like (Ubl) molecules, such as ubiquitin (Ub) and interferon-stimulating gene 15 (ISG15), which are two key regulators of the innate immune response^6^,^7^,^8^. We and others have also shown that SARS-CoV PLpro and other CoV PLpros display substantially different substrate specificities for ISG15 and certain poly-Ub chains^9^,^10^,^11^. More importantly, the DUB/deISGylating activities have been shown to play an important role in antagonizing host innate immune responses to promote viral replication^8^,^12^,^13^ although the precise roles for each activity in this antagonism have yet to be determined.

Interestingly, the CoV RNA genome encodes for two Ubl domains within nsp3 that are denoted as Ubl1^14^ and Ubl2^5^ according to their location in the nsp3 multi-domain protein. The Ubl2 domain of SARS-CoV, previously named the Ubl, was first identified by our lab through X-ray structural studies where it was found to reside directly adjacent to the N-terminus of the PLpro catalytic domain^5^. Since our original structure, the Ubl2 domain has been found to be conserved among CoVs to date, including MERS-CoV^15^, murine hepatitis virus (MHV)^11^, and infectious bronchitis virus (IBV)^16^. However, the functional roles for Ubl2 in viral pathogenesis and RNA replication remain enigmatic. So far, the majority of studies aimed at understanding the roles of viral Ubls in CoV replication have focused on the Ubl2 domain due to its location in the RNA genome and potential to modulate the enzymatic activity of PLpro. For example, we investigated the function of Ubl2 in SARS-CoV^7^ and MHV^17^ and found that the Ubl2 fold is crucial for maintaining PLpro structural integrity *in vitro*. Interestingly, in cell-based assays, SARS-CoV PLpro without its Ubl2 domain was no longer able to antagonize the pathways involved in the host innate immune response and thereby act as an IFN antagonist^7^. However, the mechanism that leads to this loss of function is not well understood because the enzyme retained its protease and DUB activities. In the case of MHV, a single point mutation in the Ubl2 domain was found to reduce the thermal stability of the papain-like protease 2 (PLP2) domain rendering the enzyme DUB deficient in cells^17^. When this mutation was inserted back into the virus, the mutant virus was attenuated in infected mice with the ability to replicate and induce a protective immune response against wild-type virus. This study revealed that the Ubl2 domain could be used as a strategy to attenuate CoV pathogenesis leading us to further investigate the function of the Ubl2 domain in MERS-CoV.

Unexpectedly, our recent cell-based studies using different truncated forms of MERS-CoV Ubl2 (MERS PLpro-∆Ubl2) suggest that the Ubl2 domain might not be as pivotal for PLpro enzyme activity as originally thought. In these cell-based assays, MERS-CoV PLpro-∆Ubl2 appeared to retain its multiple enzymatic functions and unlike SARS-CoV PLpro-∆Ubl2, preserved its ability to act as an IFN antagonist^9^. This intriguing discovery engendered the hypothesis for this study that the MERS-CoV Ubl2 domain is not required for PLpro catalytic function and stability *in vitro*. Here, we report a series of X-ray structural and kinetic studies on the MERS-CoV PLpro-ΔUbl2 construct to elucidate the importance of Ubl2 domain in MERS-CoV stability, substrate specificity and enzymatic catalysis as well as to evaluate the efficacy of a reported MERS-CoV PLpro inhibitor^18^.

## Results

### X-ray Structure Determination of MERS-CoV PLpro without the N-terminal flanking Ubl2 domain

**Figure 1:**
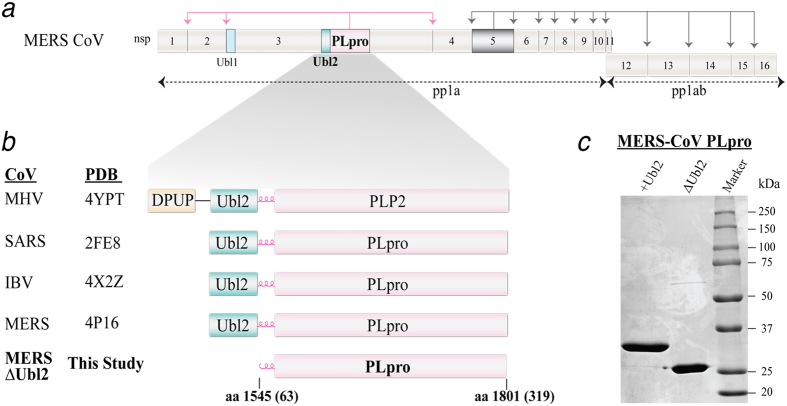
MERS-CoV polyprotein organization and design rationale for the MERS-CoV PLpro-ΔUbl2 construct of nsp3. (**a**) Non-structural proteins (nsps) are numbered 1-16 within the MERS-CoV viral polyprotein 1a and 1ab. MERS PLpro is colored in pink in nsp3 and 3CLpro, which is in nsp5, is colored in gray and their respective cleavage sites are colored accordingly and indicated by arrows. The Ubl1 (light blue) and Ubl2 (green) domains of nsp3 are indicated. (**b**) Summary of the current PLP X-ray structures and the smallest catalytic unit determined in this study. The PDB codes of the X-ray structures that were determined first for MHV PLP2, SARS-CoV, IBV and MERS-CoV PLpro, all containing the Ubl2 domain, are indicated. (**c**) SDS-PAGE (12.5%) analysis of purified MERS-CoV PLpro-Ubl2 (36 kDa) and −ΔUbl2 (29 kDa) used for activity assays and crystallization. The proteins are estimated to be >95% purity by densitometry.

Since the functionality of MERS-CoV PLpro was observed to be independent of the Ubl2 domain in cellular assays, we sought to determine the structure of the segregated catalytic core. The designed MERS-CoV PLpro-ΔUbl2 plasmid expresses only the catalytic core of PLpro (Fig. 1b). The 60 amino acids encoding for the Ubl2 domain at the N-terminus and a single cysteine from the C-terminus of PLpro were removed. The cysteine was removed to aid in crystallization. PLpro-ΔUbl2 was expressed and purified using nearly identical conditions and procedures as those for MERS-CoV PLpro flanked with the N-terminal Ubl2 domain. The MERS-CoV PLpro-ΔUbl2 catalytic domain remained stable throughout purification with minimal precipitation and no activity loss. The resulting molecular weights for MERS-CoV PLpro-Ubl2 and PLpro-ΔUbl2 after removal of the octa-histidine tags were ~36 kDa and ~29 kDa, respectively (Fig. 1c).

**Figure 2:**
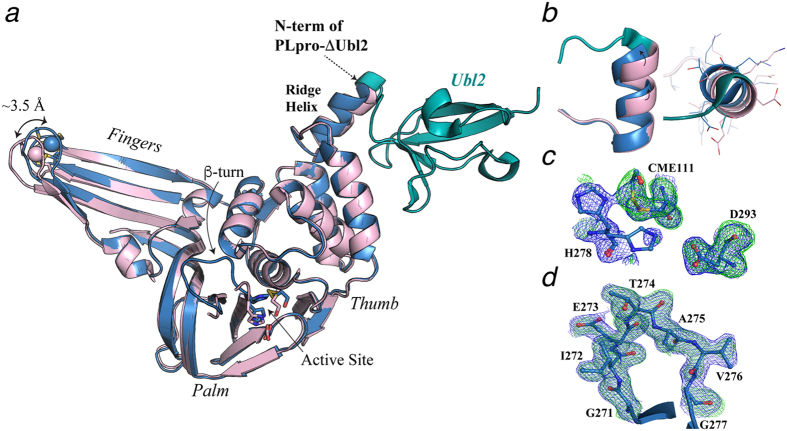
X-ray crystal structure of MERS-CoV PLpro-ΔUbl2 (blue, PDB code 5KO3) superimposed with the MERS-CoV PLpro (pink, PDB code 4P16). (**a**) Overall structure of PLpro with its three subdomains and active site labeled. The Ubl2 domain of MERS-CoV PLpro is colored in green and the zinc atom of the fingers domain is shown as a sphere with zinc-coordinating cysteines represented as sticks. The arrow indicates the difference in the position of the zinc atom between the two structures. The ridge helix is indicated, and the position of where the Ubl2 domain was truncated at N-terminus of PLpro-ΔUbl2 is indicated with an arrow. The β-turn-substrate-binding loop of MERS-CoV PLpro-ΔUbl2, observed in this study, is also indicated as ‘β-turn’. (**b**) Magnified views (elongated-left and helical wheel projection-right) of the ridge helix show the similarity between the two structures. (**c**) Electron density maps associated with the catalytic triad residues (Cys111, His278, Asp293) and β-mercaptoethanol (CME111) are shown in green mesh (F_o_-F_c_) and blue mesh (2F_o_-F_c_). F_o_-F_c_ electron density omit maps, where the catalytic triad residues were omitted from the calculations, are contoured to 3σ. Final 2F_o_-F_c_ maps are contoured to 1σ. His278 was observed to reside in two positions after occupancy refinement – one at slightly higher occupancy (0.55). (**d**) Electron density maps associated with substrate-binding loop (residues 271-277) containing the β-turn are shown in green mesh (F_o_-F_c_) and blue mesh (2F_o_-F_c_) and are contoured to 3σ and 1σ, respectively. The entire loop could be modeled into the observable density and is represented as sticks. Atoms in Panels c-d are colored as follows; nitrogens (dark blue), oxygens (red), sulfur (yellow), carbons (light blue).

PLpro-ΔUbl2 crystallized in space group P 1 2 1 with one biologically active monomer in the asymmetric unit. X-ray data were collected to 1.95 Å, and the final X-ray data collection and refinement statistics are summarized in Table 1. The final X-ray structural model for MERS-CoV PLpro-ΔUbl2 has R-values of R_work_ = 16.6% and R_free_ = 18.8%. The MERS-CoV PLpro-ΔUbl2 structure contains only the catalytic domain of PLpro with its three subdomains: thumb, fingers, and palm (Fig. 2a). The secondary structure arrangement of the catalytic domain is identical to the structure described in ref. 15 with seven α-helices (six in the thumb domain, one in the fingers domain), fourteen total β-strands (four in the thumb domain, four and two partial strands in the fingers domain, and four and two partial strands in the palm domain), and one 3_10_-helix (η) in the fingers domain.

Similar to the MERS PLpro-Ubl2 structure, the catalytic Cys111 in the MERS-CoV PLpro-ΔUbl2 structure is also modified by β-mercaptoethanol (BME) with a partial occupancy of 0.83 (Fig. 2c)^15^. Weak electron density for the catalytic His278 is observed in final F_o_-F_c_ omit maps (Fig. 2c). Due to the partially modified Cys111, His278 is observed to occupy at least one alternative position in order to accommodate the bulky, modified Cys111 residue. On the other hand, and in contrast to previously reported unbound MERS-CoV PLpro structures, including Protein Data Bank (PDB) codes 4P16^15^, 4PT5 (unpublished), 4REZ^13^ and 4RNA^18^, strong (>3σ in F_o_-F_c_ maps) and well-defined electron density is observed for the flexible loop encompassing residues 271–277 in the MERS-CoV PLpro-ΔUbl2 structure (Fig. 2d). The substrate-binding loop has been observed in the Ub-bound MERS-CoV PLpro structures^13^,^19^. As a result, we were able to readily build and refine the entire loop that is responsible for substrate binding and inhibitor recognition in CoV PLpros and PLP2s^9^,^20^. Altogether, the X-ray structural data suggests that the catalytic domain of MERS-CoV PLpro is highly stable in the absence of the Ubl2 domain.

### Structural Comparison of PLpro with and without the Ubl2 domain

There are currently four structures of MERS PLpro flanked with the N-terminal Ubl2 domain that have been determined in the absence of any bound ligand^13^,^15^,^18^. To determine if the Ubl2 domain elicits an effect on the conformation of the MERS-CoV PLpro catalytic core, we superimposed the structures of PLpro-ΔUbl2 and PLpro-Ubl2 (Fig. 2a,b). The MERS-CoV PLpro catalytic domain is observed to adopt a conformation that is nearly identical to the structure with the Ubl2 domain intact. The resulting root-mean-square-deviation (RMSD) is 0.4 Å when the C_α_ of 254 residues in PLpro-ΔUbl2 are aligned with C_α_ of 258 residues in PLpro-Ubl2. The catalytic triad, Cys111-His278-Asp293, aligns well for both enzymes except for the His occupying the conformation near the modified cysteine group. Truncation of the Ubl2 domain appears to cause only slight deviations in the ridge helix of the thumb domain due to the loss of two helix residues, Thr61 and Ala62. We also observe some variation in the position of the zinc atom in the fingers domain, which is reminiscent of the open and closed conformations observed in the Ub-bound complex^13^. This observation suggests that the zinc-fingers binding motif has high flexibility, which may provide an explanation as to why there is weaker electron density and increased B-factors associated with this region that includes residues 225–230.

### Enzymatic Activity of MERS-CoV PLpro is unaffected by the loss of the Ubl2 domain

To determine if the enzymatic activity of PLpro is dependent on the Ubl2 domain, we determined the steady-state kinetic parameters of the PLpro-ΔUbl2 and PLpro-Ubl2 catalyzed hydrolysis of three different Ub-based substrates, Ub-AMC, ISG15-AMC and Z-RLRGG-AMC, which are commonly used to assess PLpro DUB, deISGylating and proteolytic activities. The kinetic assays for each substrate were performed side-by-side with each enzyme in the same assay plates under identical assay conditions. The kinetic response of each enzyme to increasing concentrations of substrate are shown in Supplementary Fig. S1. We were unable to reach saturation with the Z-RLRGG-AMC peptide substrate up to concentrations of 75 μM, a concentration that begins to approach the concentration range whereby the inner filter effect for the AMC fluorophore can confound the assay^9^. Therefore, this first-order range of the kinetic data were fit to a line to obtain the slope, which is the apparent k_cat_/K_m_ or catalytic efficiency. In contrast, both enzymes could be saturated with ISG15-AMC and Ub-AMC substrates, and the kinetic data were fit to the Michaelis-Menten equation to obtain individual *k*_cat_ and K_m_ values. The resulting kinetic parameters for both enzymes against all three substrates are summarized in Table 2.

As suggested by our previous work in cells, the kinetic parameters for PLpro-Ubl2 and PLpro-ΔUbl2 catalyzed hydrolysis of Ub-AMC, ISG15-AMC and Z-RLRGG-AMC substrates are nearly identical for the two enzymes. Compared to the *k*_cat_/K_m_ value for the Z-RLRGG-AMC peptide, both enzymes hydrolyze Ub-AMC (~1,600 times) and ISG15-AMC (~5,000 times) more efficiently. MERS-CoV PLpro with and without the Ubl2 domain hydrolyzes ISG15-AMC substrate ~3-fold more efficiently than Ub-AMC although the turnover numbers, *k*_cat_, are identical. The higher catalytic efficiency is mainly due to the lower K_m_ value observed for ISG15-AMC. Assuming that the K_d_ ≅ K_m_, PLpro may bind ISG15 3-fold tighter compared to Ub. The results of these kinetic studies support our hypothesis that the Ubl2 domain is not required for MERS-CoV PLpro DUB, deISGylating, and proteolytic activities *in vitro*.

### MERS-CoV PLpro Ub chain specificity and poly-Ub processing are not dependent on the Ubl2 domain

**Figure 3:**
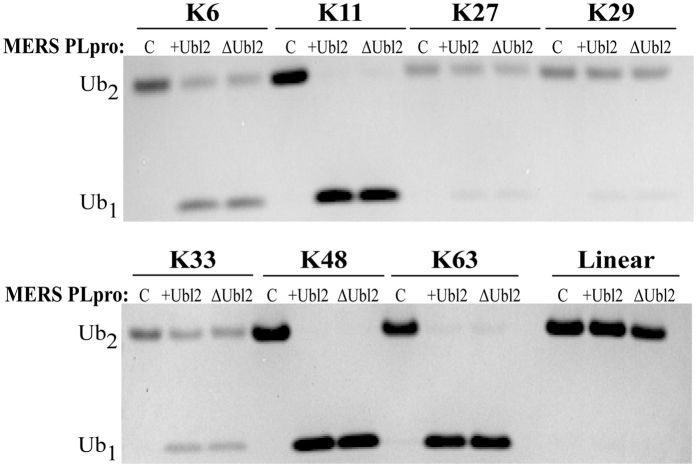
The Ubl2 domain is not required for MERS PLpro Ub_2_-processing specificity. The cleavage of different Ub_2_ linkages (Lys6, Lys11, Lys27, Lys29, Lys33, Lys48, Lys63, and linear) mediated without addition of enzyme (C) or by 160 nM PLpro-Ubl2 (+Ubl2) or PLpro-∆Ubl2 (∆Ubl2) for 2 hours analyzed by SDS-PAGE.

Recent studies demonstrated that MERS-CoV PLpro with an intact Ubl2 domain has broad Ub chain specificity based on cleavage of various diubiquitin (Ub_2_) chains with isopeptide linkages^10^. MERS-CoV PLpro has been proposed to use a single monoubiquitin recognition sub-site, S1, for Ub binding to processes all Ub chains^9^,^10^. It is possible that the MERS-CoV Ubl2 domain could function to assist PLpro in its ability to discriminate between different Ub_2_ linkages. We therefore tested the ability of MERS-CoV PLpro with and without an intact Ubl2 domain to process different Ub_2_ isopeptide linkages, including Lys6, Lys11, Lys27, Lys29, Lys33, Lys48, and Lys63, and linear Ub_2_, which is linked via the amino-terminal Met residue. We incubated each of the substrates with MERS-CoV PLpro-Ubl2 or PLpro-ΔUbl2 for 2 hours and analyzed the cleaved products by SDS-PAGE. The results are shown in Fig. 3, and they indicate that the Ubl2 domain does not impact the ability of PLpro to recognize different isopeptide linkages. MERS-CoV PLpro is capable of recognizing and processing all Ub_2_ linkages except the peptide linkage (linear). Linkages that were efficiently cleaved to nearly all monoubiquitin after the time course were Lys11, Lys48, and Lys63. However, linkages with partially reacted or unreacted Ub_2_ species were Lys6, Lys27, Lys29, and Lys33. These results are also consistent with recent findings that MERS-CoV PLpro without the Ubl2 domain was still able to be modified by a K48-linked Ub_2_ warhead supporting the hypothesis that the Ubl2 domain is not involved in substrate recognition^10^.

**Figure 4:**
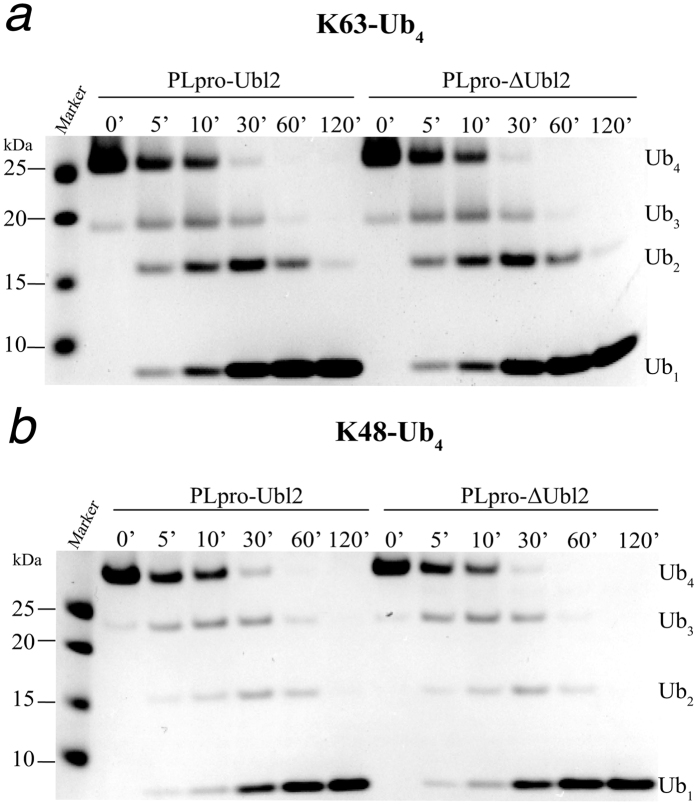
Ubl2 domain is not required for MERS PLpro Ub_4_ processing. (**a**,**b**) Time course of cleavage processing of Lys63-Ub_4_ (**a**) and Lys48-Ub_4_ (**b**) by 160 nM PLpro-Ubl2 and PLpro-∆Ubl2. Samples were quenched with sample buffer at the indicated time points and products were analyzed by SDS-PAGE. The negative control (time point ‘0’) was incubated without addition of enzyme.

Since Lys48- and Lys63-linked polyubiquitin chains are preferentially utilized in host innate immune response pathways^21^,^22^, we further evaluated the kinetics of hydrolysis of Lys63- and Lys48-linked tetraubiquitin (Ub_4_) chains by PLpro-Ubl2 or PLpro-ΔUbl2 to evaluate if the Ubl2 domain is involved higher-order polyubiquitin chain processing. The cleavage assay was performed over a 2 hour time course, and reaction products were analyzed at various time points from 5 minutes to 2 hours using SDS-PAGE (Fig. 4a,b). Ub_4_ substrates without addition of enzyme served as the negative control. The cleavage assays for Lys63-Ub_4_ (Fig. 4a) and Lys48-Ub_4_ (Fig. 4b) are nearly identical for the reactions catalyzed by MERS-CoV PLpro with and without the Ubl2 domain. Both substrates are readily converted into monoubiquitin species after 2 hours with no apparent accumulation of other Ub forms consistent with previous reports^9^,^10^. Together, the results suggest that the Ubl2 domain of MERS-CoV PLpro is not involved in any significant recognition and cleavage of polyubiquitin chain substrates.

### MERS-CoV PLpro-ΔUbl2 is thermally stable

Although MERS-CoV PLpro-ΔUbl2 and MERS-CoV PLpro have nearly identical substrate recognition patterns and kinetic parameters at room temperature, it is possible that the Ubl2 domain may alter the thermostability of the catalytic domain at higher temperatures. To test this possibility, we performed circular dichroism (CD) melting studies on each enzyme by monitoring the CD signal as a function of temperature and then determining the thermal melting temperatures (T_m_). The average T_m_ values from three independent experiments were found to be 61.2 ± 0.3 °C for MERS-CoV PLpro-Ubl2 and 60.7 ± 0.2 °C for PLpro-ΔUbl2 (data not shown). The change in thermal melting temperature (ΔT_m_) of only 0.6 °C indicates that MERS-CoV PLpro is a structurally stable enzyme even in the absence of the N-terminal Ubl2 domain. In fact, MERS-CoV PLpro-ΔUbl2 remains folded at higher temperatures compared to MHV PLP2, which unfolds at temperatures less than 50 °C^17^. Therefore, we conclude that the MERS-CoV Ubl2 domain does not stabilize or destabilize the catalytic domain *in vitro*.

### Evaluation of compound F2124–0890, a purported inhibitor of MERS-CoV PLpro

Recent studies by Lee *et al*. reported that compound **4**, commercial code **F2124–0890** (Life Chemicals), inhibits MERS-CoV and SARS-CoV PLpro activity with IC_50_ values in the low micromolar range^18^. We performed an independent analysis of the ability of compound **F2124–0890** to inhibit MERS-CoV PLpro and PLpro-ΔUbl2 in addition to other viral and cellular USPs. First, we varied concentrations of compound **F2124–0890** and measured the percent inhibition of viral papain-like proteases, including MERS-CoV PLpro-Ubl2, PLpro-ΔUbl2, SARS-CoV PLpro and MHV PLP2 both in the absence and presence of 5 mM DTT, a reducing agent (see Supplementary Fig. S2). Compound **F2124–0890** equally inhibits both MERS-CoV PLpro-Ubl2 and PLpro-ΔUbl2 both in the absence and presence of reducing agent and supports our general observation that the Ubl2 domain does not influence MERS-CoV PLpro catalytic function. The resulting IC_50_ values are given in Table 3.

Next, we performed the same experiment with 3 different human USPs, USP7, USP17 and USP28, both in the absence and presence of reducing agent (5 mM DTT). What is immediately apparent from the data presented in Supplementary Fig. S2 is that compound **F2124–0890** is capable of significant inhibition of all of the viral and human USP enzymes but only in the absence of reducing agent. In the presence of reducing agent, the inhibition of these enzymes is either eliminated or significantly reduced. Our results are summarized in Table 3, and they stand in strong contrast to those obtained by Lee *et al*. who reported that compound **F2124–0890**, compound **4** in their studies, strongly inhibits MERS-CoV (IC_50_ = 6.2 μM) and SARS-CoV (IC_50_ = 10.9 μM) PLpro in the presence of reducing agent, 5 mM DTT or 2 mM GSH^18^. Another striking observation from the data presented in Table 3 is that compound **F2124–0890** is non-selective, i.e. it is promiscuous, potently inhibiting multiple USP homologs.

**Figure 5:**
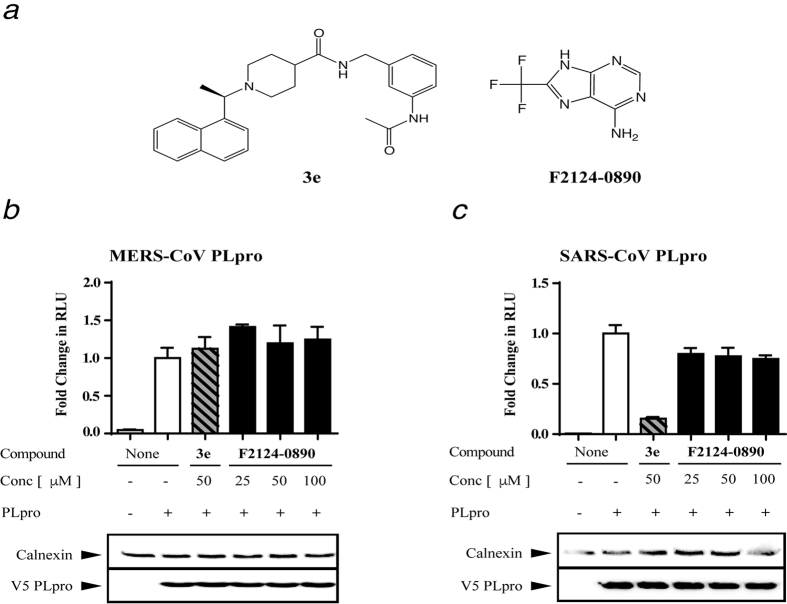
Luciferase-based biosensor assay reveals lack of inhibitory potency of F2124-0890 against MERS-CoV and SARS-CoV PLpro. (**a**) Structure of SARS-CoV PLpro inhibitor **3e** and purported inhibitor **F2124-0890**. (**b**,**c**) A biosensor endpoint assay was used to determine if compound **F2124–0890** inhibits SARS- or MERS-PLpro activity in cells. The graph shows representative results of each compound with error bars depicting standard deviation. Relative expression of PLpro and the cellular protein calnexin were determined by western blot using monoclonal antibodies specific for V5 (PLpro) and calnexin. Each compound concentration was evaluated in triplicate and experiments were performed three times.

We further evaluated **F2124–0890** using a recently described cell-based assay, named the pGlo biosensor assay, for CoV PLpro activity^23^. HEK-293T cells were transfected with plasmid DNA expressing an inactive form of luciferase and either the wild-type or a catalytic mutant (Cys111Ala) of PLpro. We found that expression of SARS-CoV and MERS-CoV PLpro activate the biosensor and that a selective SARS-CoV PLpro inhibitor that we developed, compound **3e**^20^,^23^, blocks SARS-CoV PLpro activity. In contrast, addition of **F2124–0890** had no effect on either MERS-CoV (Fig. 5b) or SARS-CoV (Fig. 5c) PLpro activity in the biosensor assay, which supports and confirms our *in vitro* results that compound **F2124–0890** loses potency in physiological reducing environments.

## Discussion

We investigated the function of the Ubl2 domain of MERS-CoV PLpro in substrate recognition and catalysis, structural stability and inhibition by a purported small molecule inhibitor. We also report the first X-ray crystal structure of a CoV PLpro or PLP2 without its flanking Ubl2 domain. We found using X-ray crystallography that removal of the Ubl2 domain from MERS-CoV PLpro did not alter the structure of the catalytic domain significantly nor did it change the structural stability as determined by melting temperatures derived from CD melting curves. Steady-state kinetic studies revealed that the Ubl2 domain associated with MERS-CoV PLpro is not required for its enzymatic function, including DUB, deISGylating, and proteolytic activities. In addition, examining MERS-CoV PLpro-mediated catalysis towards different polyubiquitin substrates with different isopeptide linkages revealed that the Ubl2 domain does not influence high-order Ub chain processing or Ub chain specificity. Overall, these studies indicate that the core catalytic domain of MERS PLpro is a robust enzyme that can be used in cell-based and *in vitro* assays making it highly amenable for high-throughput screening to evaluate potential inhibitors.

Whether other domains within the MERS-CoV nsp3 participate in the discrimination of different Ub chain linkages still remains unclear. Our findings suggest, however, that the Ubl2 domain is likely not involved in this function. MERS-CoV PLpro is capable of cleaving a variety of different chain linkages, though some chains are more favored. The substrate recognition of MERS-CoV PLpro is therefore similar to MHV PLP2^11^. Our findings support that the recognition and cleavage of these Ub_2_ chains by MERS-CoV PLpro is independent of the presence of the Ubl2 domain.

MERS-CoV PLpro can also hydrolyze both Lys48- and Lys63-linked polyubiquitin chains to monoubiquitin at equal rates either with or without the Ubl2 domain. The rapid processing of both chains to monoubiquitin suggests that MERS-CoV PLpro, similar to MHV PLP2, utilizes monoubiquitin recognition at a single S1 sub-site to cleave all Ub chains and ISG15. In contrast, SARS-CoV PLpro prefers to utilize Ub_2_ recognition to hydrolyze substrates. For example, SARS-CoV PLpro hydrolyzes Lys48-linked chains more efficiently by using two Ub binding sites across S2-S1 sub-sites as opposed to Lys63-linked chains, which are recognized by the single S1 sub-site^8^. The structural basis for Ub_2_ recognition was recently revealed via the crystal structure of SARS-CoV PLpro in complex with Lys48-linked Ub_2_ supporting the previous models^8^,^9^,^10^,^24^. In the S2 binding pocket, the ridge helix of SARS-CoV PLpro, which is immediately adjacent to the Ubl2 domain, actively engages with the distal Ub of the K48-linked Ub_2_ substrate^24^. We propose that the ridge helix of MERS-CoV PLpro may not actively participate in substrate binding as with SARS-CoV PLpro. However, we can only conclude from the two residues removed from the ridge helix that these specific residues are not involved in ISG15 or polyubiquitin catalysis, as no further mutagenesis was done in this region. Altogether, our findings, coupled with the aforementioned studies, clearly show that MERS-CoV and SARS-CoV PLpro utilize different mechanisms when recognizing and cleaving host proteins, and specifically, MERS-CoV PLpro likely does not contain the S2 sub-site.

Our original X-ray structure of SARS-CoV PLpro revealed for the first time the presence of Ubl domains in CoV nsp3s, and it established the first known vUSP defining a new class^5^. This seminal work on vUSPs set the stage for new discoveries, including a bioinformatics study on the homologies of human USPs which revealed that the Ubl fold, resembling the β-grasp architecture of Ub, is predicted to be present in at least 16 human USPs^25^. The locations of these Ubl domains can be found at either their N- and C-termini or embedded within their catalytic core. A plethora of Ubl domains residing within human USPs suggest that they may play a significant functional role in the tightly orchestrated process of protein degradation as well as other critical signaling processes. Thus far, the function of Ubl domains in USPs have been attributed to the alteration of enzyme catalysis and specificity, or the recruitment of binding partners to mediate processes, including cellular localization, trafficking, and signal transduction^25^.

Although extensive studies have characterized Ubl domains from human USPs, the function of viral Ubl domains in biological systems remains elusive. Initially, the function of the Ubl2 domain was evaluated in cell-culture with SARS-CoV PLpro where the Ubl2 domain was found to be essential for PLpro’s ability to act as an IFN antagonist and inhibit IRF3 phosphorylation or NF-κB signaling^7^. Interestingly, when the Ubl2 domain was removed from PLpro, SARS-CoV PLpro-ΔUbl2 maintained its DUB and protease activity. However, further characterization of SARS-CoV PLpro-ΔUbl2 *in vitro* was not pursued due to its instability during expression and purification. Interestingly, in a recent study both SARS-CoV and MERS-CoV PLpro-ΔUbl2 constructs were expressed and purified *in vitro*. Both enzymes maintained their catalysis towards a K48-linked Ub_2_ warhead as compared to PLpro with the Ubl2 domain^10^. These results suggest that the Ubl2 domain may not be involved in the mechanism of substrate recognition or catalysis for PLpros. However, the Ubl2 domain was found to be important for MHV PLP2 thermal stability^17^. Previous work evaluated the effect of a conserved single Val787Ser mutation in the Ubl2 domain, which was found to decrease PLP2 enzymatic activity at physiological temperatures and attenuate mutant virus in mice. We propose that a similar mutation could provoke an “unraveling effect” if introduced in other Ubl2 domains adjacent to PLpro. However, in the case of MERS-CoV PLpro, we show that we can completely remove the Ubl2 domain of MERS-CoV PLpro, and the resulting PLpro catalytic core is still able to maintain its stability and catalysis *in vitro* and in cells^9^.

We also investigated whether a compound, **F2124–0890**, which was recently reported to be a potent and selective inhibitor of both MERS-CoV and SARS-CoV PLpro^18^, could also inhibit MERS-CoV PLpro without its Ubl2 domain. This purine analogue was first synthesized in 1958 as a potential anticancer agent, and in the late 1980s and early 1990s, the compound was used as a reactant for designing arrhythmia and antiviral drugs as well as compounds set to regulate plant growth^26^,^27^. For over 20 years, **F2124–0890** was seldom reported in the literature until in 2014 when it was identified as an inhibitor of SARS-CoV 3CLpro by Lee *et al*.^28^. In that study, **F2124–0890**, referred to as compound **14**, was identified as an inhibitor of SARS-CoV 3CLpro via a high-throughput screen. **F2124–0890** was found to inhibit SARS-CoV 3CLpro with mixed-type inhibition (IC_50_ of 13.9 μM). In 2015, Lee *et al*. also performed a similar HTS study this time against MERS-CoV and SARS-CoV PLpro and they identified the same compound, **F2124–0890**, which they referred to in that study as compound **4**. It was reported that compound **4** inhibited PLpro from both CoVs with low micromolar IC_50_ values and, based on the mechanisms of inhibition of each enzyme, was predicted to act as a competitive inhibitor against MERS-CoV PLpro and an allosteric inhibitor of SARS-CoV PLpro. The binding mechanism was described to take place at either the active site pocket or an unknown allosteric site.

In contrast to the aforementioned studies of Lee *et al*., we demonstrate that **F2124–0890** (a.k.a. compound **4** or **14**) is non-selective under non-reducing conditions; inhibiting all viral and human cysteine proteases tested and confirming that it is a pan-assay interference compound (PAIN)^29^. We also evaluated the inhibitory ability of **F2124–0890** under reducing conditions by either placing DTT in the biochemical assays or using the natural reducing environment of the cell. We found that reducing agent either greatly diminished or eliminated the ability of **F2124–0890** to inhibit MERS-CoV and SARS-CoV PLpro and the USPs tested. Possible explanations as to why the compound may only show efficacy under non-reducing conditions could be that the compound binds in a non-specific manner to viral and human USPs and promotes reversible-oxidation of the active site cysteine. Another explanation could be that chemical reducing agents may directly compete against the inhibitor for binding to the active site. Reducing agents, such as DTT and BME, can modify active site cysteines. The bulky modified cysteine formed by the BME reducing agent observed in the crystal structure supports the fact that the inhibitor may not be able to bind under the reducing conditions due to the encumbered active site pocket. However, **F2124–0890** is unable to inhibit MERS-CoV or SARS-CoV PLpro under the natural reducing conditions of a cell indicating that inhibition by this compound is complex and likely non-specific. It is clear from our data and the data presented in the literature that **F2124–0890** has poor selectivity among cysteine proteases, lacks inhibitory potency in cell-based assays, and has greatly reduced or no inhibitory potency in *in vitro* assays in the presence of reducing agents. Therefore, **F2124–0890** is likely a PAIN that should not be pursued further as a lead compound for therapeutic development or other uses.

In summary, the catalytic core of MERS-CoV is stable and highly active without its Ubl2 domain. MERS PLpro-∆Ubl2 exhibits the same substrate specificity profile of MERS-CoV with an intact Ubl2 domain suggesting that the Ubl2 domain is not necessary for normal MERS-CoV PLpro function. MERS PLpro-∆Ubl2 is highly amenable to enzyme inhibitory studies, and it easily forms crystals that diffract to high resolution. Overall, the properties of MERS PLpro-∆Ubl2 suggest that it may be an ideal construct for structure-based inhibitor design efforts.

## Methods

### Expression and Purification of MERS-CoV PLpro-Ubl2 and PLpro-ΔUbl2

The genes encoding MERS-CoV PLpro-UBL and PLpro-ΔUbl2 were previously cloned into pEV-L8 expression vector^9^. A stop codon was incorporated at the C-terminal end (before residue C320) of PLpro-ΔUbl2 by site-directed mutagenesis. The resulting pEV-L8-PLpro-ΔUbl2 (Fig. 1b) plasmid DNA was subjected to DNA sequencing using the Purdue Genomics Core Facility to confirm that the correct construct was generated.

Two liters of Super Broth media^9^ was inoculated with 10 ml of *E. coli* BL21 (DE3) cells transformed with either pEV-L8-PLpro-Ubl2 or pEV-L8-PLpro-ΔUbl2. After centrifugation (6,750 g, 4 °C, 20 minutes), the harvested cells (16–18 g) were frozen at −80 °C. On the day of purification, the frozen cell pellets were thawed and then resuspended in 74 ml buffer A (20 mM Tris, pH 7.5, 500 mM NaCl, 5 mM imidazole, 10 mM BME, and 5% glycerol) supplemented with 0.25 mg/ml lysozyme and 10 μg/ml DNase I. The resuspended cells were lysed on ice by sonication using a Branson Digital Sonifier (70% amplitude; 15 minutes, 10 s pulses, 10 s delays). The lysed cell debris was then removed by centrifugation (27,200 g, 4 °C, 60 minutes).

The clarified lysate was then loaded onto a 5 ml HisTrap FF column (GE Healthcare) that was pre-charged with Ni^2+^ and equilibrated with 3% buffer B (20 mM Tris, pH 7.5, 500 mM NaCl, 400 mM imidazole, 10 mM BME, and 5% glycerol). After washing unbound proteins with 5 column volumes (25 mL) of 3% buffer B, PLpro was eluted with a 110 ml linear gradient (3% to 100%) of buffer B at a flow rate of 3 ml/min. The His_8_-tag was then removed by addition of tobacco etch virus (TEV) protease (1 mg TEV: 6 mg PLpro). After incubating for 4 hours at 25 °C and then 4 °C overnight, the enzyme was passed over the same HiTrap column and the unbound, untagged PLpro was collected in the flow-through. Untagged PLpro was then concentrated to 2–5 mg/ml using an Amicon Ultrafiltration Centrifugal device (10 kDa MW cutoff) while buffer exchanging into S75 buffer (10 mM Tris, pH 7.5, 100 mM NaCl, 10 mM DTT, and 5% glycerol). The final purified protein (Fig. 1c) was flash-frozen with liquid nitrogen in 100 μl aliquots and stored at −80 °C.

### Crystallization and X-ray Structure Determination of MERS PLpro-ΔUbl2

For crystallization, the frozen, untagged PLpro was thawed on ice and loaded onto a HiLoad 26/60 Superdex 75 column (GE Healthcare) equilibrated with S75 buffer. Fractions containing active PLpro were then pooled and concentrated to 15 mg/ml for crystallization. A screen for initial crystallization conditions was performed using a Mosquito^®^Crystal liquid handling robot (TTP Labtech) in sitting drop mode and a series of sparse-matric crystallization screens (Qiagen). Sitting drops were prepared by adding 100 nl of purified MERS-CoV PLpro-ΔUbl2 to 100 nl of reservoir solution. Three protein concentrations (5 mg/ml, 10 mg/ml, and 15 mg/ml) were setup in each of the three sub-wells in a 96–3 well sitting drop vapor diffusion plates (Greiner CrystalQuick crystallization plate). An initial crystallization hit from the cation suite containing 4.5 M Ammonium acetate and 0.1 M Tris-HCl, pH 8.5 was observed with Rigaku Minstrel^®^ HT imaging robot after 1 week of incubation at 20 °C. Further optimization at 4 °C with drops containing 2 μl of purified MERS-CoV PLpro-ΔUbl2 at 10 mg/ml and 2 μl reservoir (5.5 M Ammonium Acetate, 0.1 M Tris-HCl, pH 8.5) yielded crystals with approximate dimensions of 0.05–0.1 mm after one week. Crystals were harvested using pins with nylon loops, transferred briefly to a cryo-protectant solution containing reservoir solution that was supplemented with 20% glycerol and then immediately flash-cooled by plunging into liquid nitrogen. Crystals were placed into SPINE pucks for transport to the Advanced Photon Source Synchrotron (APS), Argonne National Laboratory (ANL). X-ray data were collected on crystals using beamline 21-ID-F at the Life Sciences-Collaborative Access Team (LS-CAT).

X-ray data were collected on a single MERS-CoV PLpro-ΔUbl2 crystal using 1° rotations at 100 °K. X-ray data were indexed, processed, and scaled using HKL2000^30^. To determine the initial phases for the structure, molecular replacement with Phaser was performed using the structure of MERS-CoV PLpro-Ubl2 apo (PDB code 4P16) as a search model^15^. Model building and refinement on the resulting structural solution containing one molecule in the asymmetric unit was completed using Coot^31^ and Phenix Refine^32^ using stimulating annealing for initial refinements to limit bias. Final data collection statistics and refinement parameters are shown in Table 1. Figures were generated with PyMOL (The PyMOL Molecular Graphics System, 1.8.0 Schrödinger, LLC).

### Steady-State Kinetic Studies

The kinetic parameters of PLpro-Ubl2 and PLpro-ΔUbl2 for catalyzing the reaction of Ub-AMC (LifeSensors, Inc.), ISG15-AMC (Boston Biochem/R&D Systems), and a peptide substrate, Z-RLRGG-AMC (Bachem), were determined using a modified protocol in ref. 9. The release of the fluorophore, 7-amino-4-methylcoumarin (AMC) group (λ_ex_ = 360 ± 40 nm, λ_em_ = 460 ± 40 nm) from the substrate was monitored in the form of relative fluorescence units as a function of time (RFU/min) using a BioTEK Synergy H1 multimode microplate reader at 25 °C. The concentration of Ub-AMC was varied from 0.08 μM up to 30 μM. Reactions were initiated with 5.2 nM PLpro for low Ub-AMC (<10 μM) concentrations and 1.3 nM PLpro for high concentrations (≥10 μM) to ensure initial rates were captured. For the ISG15-AMC assay, the concentration of substrate was varied from 0.02 μM to 12 μM using 0.39 nM PLpro to initiate hydrolysis. The concentration of Z-RLRGG-AMC was varied from 1.6 μM up to 75 μM initiating peptide hydrolysis with 1.0 μM PLpro. The initial rates of the reaction were converted to initial velocity (v; μM/min) using the extinction coefficient (Δε; RFU/μM) of product or the maximum amount of AMC that is released from the reaction. The reaction rates (v/[E]; min^−1^) measured in triplicate were plotted as a function of substrate concentration, [S]. For saturating substrates, kinetic parameters, k_cat_ and K_m_, were determined using the SigmaPlot (v12) enzyme kinetics module from the non-linear regression Michaelis-Menten equation (1):


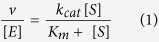


where k_cat_ is defined as the number of substrate molecules hydrolyzed by PLpro per minute per active site and K_m_ represents the substrate concentration where the reaction rate is half-maximal. From these kinetic parameters, the catalytic efficiency (k_cat_/K_m_) of the enzyme was determined. The standard deviation of the k_cat_/K_m_ was calculated using the following equation (2):


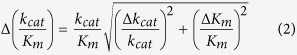


where Δk_cat_ and ΔK_m_ are the associated errors from the k_cat_ and K_m_ values, respectively. For the nonsaturating peptide substrate, the apparent k_cat_/K_m_, values were approximated using a linear regression module in GraphPAD Prism6 from the following equation (3):


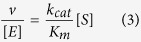


### Ub_2_ and Ub_4_ Chain Cleavage Assays

A Ub_2_ panel containing various isopeptide chain linkages (Boston Biochem) of Lys6, Lys11, Lys27, Lys29, Lys33, Lys48, Lys63, and linear Ub_2_ (UbiQ explorer planel) were incubated with 160 nM PLpro-Ubl2 or PLpro-ΔUbl2 at 25 °C for 2 hours in reaction buffer (50 mM HEPES, pH 7.5, 150 mM NaCl, 5 mM DTT). Reactions without enzyme served as a negative control. After 2 hours reaction mixtures were quenched with LDS sample buffer (Life Technologies) and then loaded onto a gradient (4–12%) SDS-PAGE at Ub_2_ concentrations of 0.5 μg and 1.5 μg per well for Lys6-Lys63 and linear, respectively. Ub_4_ cleavage assays for Lys48- and Lys63-linked chains (LifeSensors, Inc.) were performed using the same enzyme concentrations, buffer compositions, and negative control as described above. The reaction was quenched at five different time points from 5 minutes to 2 hours and analyzed by SDS-PAGE loaded at 0.5 μg per well.

### CD melting studies

Protein samples at 1–2 μM were loaded in a 10 mm quartz cell (Starna Cells) with magnetic stir bar in 2.5 ml of 0.1 M potassium phosphate (pH 7.5). The CD signal was measured at 220 nm as the temperature was increased at a step interval of 0.4 °C and rate of 1.0 °C/min as proteins were denatured using a Chirascan circular dichroism (CD) spectrometer (Applied Photophysics) equipped with a temperature control bath (Quantum Northwest Inc.). The average T_m_ for each enzyme was calculated from three independent experiments by determining the maxima of the first derivative peak using SigmaPlot (v12).

### Determination of IC_50_ Values for inhibitors under Reducing and Non-reducing conditions

The exact compound **4** investigated by Lee *et al*. was purchased from Life Chemicals, Inc. (CAS # 2993–05–7), referred to as the company code name (**F2124–0890**) in this study^18^. Inhibition assays were performed in the presence and absence of 5 mM DTT for vUSPs, MERS-CoV PLpro, SARS-CoV PLpro, and MHV PLP2 (400 nM, 20 nM, and 3 μM final enzyme concentrations), at a 100 μL scale and three human USPs, USP7, USP17, and USP28 (1 nM, 5 nM, and 10 nM final enzyme concentrations), at a 30 μl scale. Assay conditions for PLpro-ΔUbl2 resembled those used for MERS-CoV PLpro containing the Ubl2 domain. The enzymatic activities of SARS-CoV PLpro and MHV PLP2 were monitored with 50 μM Z-RLRGG-AMC in assay buffer (50 mM HEPES, pH 7.5, 0.1 mg/ml BSA). The enzymatic activity of MERS-CoV PLpro was monitored with 75 μM Z-RLRGG-AMC in assay buffer described by Lee *et al*.^18^. The enzymatic activities of human USPs were monitored with 0.5 μM Ub-AMC in the same buffer^18^. The inhibitor was incubated with enzymes for 5 minutes before the reaction was initiated with substrate monitoring fluorescence using the BioTEK Synergy H1 multimode microplate reader at 25 °C. For IC_50_ determination, data in non-reducing conditions were fit to the Hill equation (4):


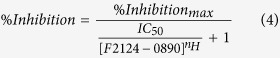


where n_H_ represents the Hill coefficient. In the presence of reducing agent, data were either fit to the Hill equation, the Michaelis-Menten equation, or if the percent inhibition was under 50% at 200 μM **F2124–0890**, it was assumed that the IC_50_ value was >200 μM.

### Biosensor assay

HEK293T cells were transfected with 150 ng pGlo-30F-RLKGG, 25 ng pRL-TK (Promega) and 150 ng of plasmid DNA expressing the indicated viral protease or empty vector. At 20 hours post-transfection, cells were lysed with 1X passive lysis buffer (Promega) and 25 μl of lysate was assayed for luciferase activity using 96-well white bottom plates (Corning) and dual luciferase activating reagents (Promega). To evaluate expression of protein, western blotting for detection of the V5-epitope-tagged protease was performed. Briefly, 25 μl of lysate was mixed with 25 μl of 2X sample buffer, and proteins were separated by electrophoresis on 10% SDS-PAGE, transferred to PVDF membrane, and probed with mouse anti-V5 (Invitrogen) as previously described^23^. HRP-conjugated goat-anti-mouse (Southern Biotech) was used as the secondary antibody with detection using the Western Lighting Chemiluminescence Reagent Plus (Perkin Elmer) and visualized using a FluoroChemE Imager.

## Additional Information

**Accession codes**: The atomic coordinates and structure factors amplitudes of MERS PLpro-ΔUBL apo have been deposited in the Protein Data Bank under accession code 5KO3.

**How to cite this article**: Clasman, J. *et al*. X-ray Structure and Enzymatic Activity Profile of a Core Papain-like Protease of MERS Coronavirus with utility for structure-based drug design. *Sci. Rep.* **7**, 40292; doi: 10.1038/srep40292 (2017).

**Publisher's note:** Springer Nature remains neutral with regard to jurisdictional claims in published maps and institutional affiliations.

## Supplementary Material

Supplementary Information

## Figures and Tables

**Table 1:** Data-collection and structure refinement statistics.

PDB entry	5KO3 (MERS PLpro-ΔUbl2)
*Data-collection parameters*	
Beamline	21-ID-F
Wavelength (Å)	0.98
Space group	*P121*
Unit cell dimensions:	
*a, b, c* (Å)	*83.7, 30.5, 86.7*
*α, β, γ* (°)	*90, 116, 90*
Resolution (Å)	100–1.95 (1.98–1.95)^a^
Number of reflections observed	351471
Number of unique reflections	29405
*R*_*merge*_ (%)^b^	7.1 (64.6)
*R*_*pim*_ (%)^c^	4.0 (35.8)
*CC*_*1/2*_ (*%) in highest shell*	77.3
CC* (%) in highest shell	93.4
*I/σI*	26.0 (2.4)
% Completeness	98.8 (98.3)
Redundancy	4.1 (4.2)
*Refinement*	
Resolution range (Å)	43.2–1.95 (2.02–1.95)
No. of reflections in working set	29042
No. of reflections in test set	1470
*R*_*work*_ (%)^d^	16.6 (20.5)
*R*_*free*_ (%)^e^	18.8 (23.3)
Wilson B factor (Å^2^)	29.5
Average B factor (Å^2^)	39.6
RMSD from ideal geometry	
Bond length (Å)	0.015
Bond angle (deg)	1.36
Ramachandran plot	
Most favored (%)	95.8
Allowed (%)	3.8
Disallowed (%)	0.4

^a^Values in parentheses are for the last (highest resolution) shell.

^b^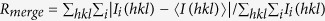, where *I*_*i*_(*hkl*) is the intensity of a given reflection, and *I*_*i*_(*hkl*) is the mean intensity of symmetry-related reflections.

^c^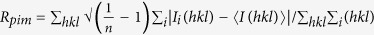, where n is the multiplicity for multiplicity-weighted R_merge_.

^d^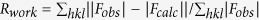, where *F*_*obs*_ and *F*_*calc*_ are the observed and calculated structure factors, respectively.

^e^*R*_*free*_ was calculated using 5% of the data set chosen at random that were excluded from the refinement.

**Table 2:** Kinetic parameters for PLpro-Ubl2 and PLpro-∆Ubl2 using three different FRET Ub-based substrates.

Enzyme	Substrate		
Kinetic Parameter	RLRGG-AMC	Ub-AMC^a^	ISG15-AMC^a^
**PLpro-Ubl2**			
k_cat_/K_m_ (μM^−1^ min^−1^)	0.003^b^	4.8 ± 0.4	13.4 ± 0.7
k_cat_ (min^−1^)	N/A	20.8 ± 0.5	21.2 ± 0.3
K_m_ (μM)	N/A	4.4 ± 0.4	1.6 ± 0.1
**PLpro-ΔUbl2**			
k_cat_/K_m_ (μM^−1^ min^−1^)	0.003^b^	4.7 ± 0.6	15.1 ± 0.8
k_cat_ (min^−1^)	N/A	21.4 ± 0.8	19.0 ± 0.3
K_m_ (μM)	N/A	4.6 ± 0.6	1.3 ± 0.1

^a^Steady-state values reported as a mean ± standard deviation, were determined from a minimum of triplicate measurements (best-fit slopes shown in Supplementary Fig. S1).

^b^Value of k_app_ with nonsaturating substrate approximates k_cat_/K_m_.

**Table 3:** Effect of Reducing agent on the Nonselective Inhibition of **F2124–0890** towards Viral and Human Proteases.

			−DTT		+DTT	
	Enzyme	Lee *et al*.^b^	IC_50_ (μM)^c^	Hill Coefficient (n_H_)	IC_50_ (μM)^a^	Hill Coefficient (n_H_)
Viral	MERS PLpro-Ubl2	6.2 ± 0.9	11.8 ± 0.4	2.5 ± 0.2	87.6 ± 24.9^c^	1.3 ± 0.2
	MERS PLpro-ΔUbl2		8.7 ± 0.3	2.6 ± 0.2	64.6 ± 10.9^c^	1.5 ± 0.2
	SARS PLpro	10.9 ± 0.9	4.9 ± 0.4	1.6 ± 0.2	—	
	MHV PLP2		18.0 ± 0.9	1.6 ± 0.1	43.1 ± 3.9^d^	
Human	USP7		3.8 ± 0.8	3.7 ± 1.5	—	
	USP17		2.7 ± 0.2	2.2 ± 0.3	>200	
	USP28		4.0 ± 0.2	2.2 ± 0.1	>200	

^a^No inhibition.

^b^IC_50_ values previously reported from ref. 18.

^c^Data fit to the Hill Equation.

^d^Data fit to the Michaelis-Menten Equation.
