# Supplementary material for: X-ray Structure and Enzymatic Activity Profile of a Core Papain-like Protease of MERS Coronavirus with utility for structure-based drug design
Source: Sci Rep. 2017 Jan 12;7:40292. doi: 10.1038/srep40292 (PMC5228125; doi:10.1038/srep40292)

Supplementary Information

X-ray Structure and Enzymatic Activity Profile of a Core Papain-like Protease of MERS Coronavirus with utility for structure-based drug design

Jozlyn Clasman[1], Yahira M. Báez-Santos[1,2], Robert C. Mettelman[3], Amornrat O'Brien[3], Susan C. Baker[3], and Andrew D. Mesecar[1,4,5,6]

[1]Department of Biological Sciences, Purdue University, West Lafayette, IN
[2]Current Address: Weldon School of Biomedical Engineering, Purdue University, West Lafayette, IN
[3]Department of Microbiology and Immunology, Loyola University Chicago, Stritch School of Medicine, Maywood, IL
[4]Department of Biochemistry, Purdue University, West Lafayette, IN
[5]Center for Cancer Research, Purdue University, West Lafayette, IN

Figures

Supplementary Figure S1. The kinetic response of MERS-CoV PLpro-Ubl2 (black circles) and PLpro-ΔUbl2 (white circles) to the increasing concentrations of three different ubiquitin-based substrates. (a) RLRGG-AMC. (b) ISG15-AMC. (c) Ub-AMC. Data in panel a failed to reach saturation and were therefore fit to a line whereas panels b and c were fit to the Michaelis-Menten equation. All data were measured in triplicate, and the error bars represent the standard deviations from the triplicate data.

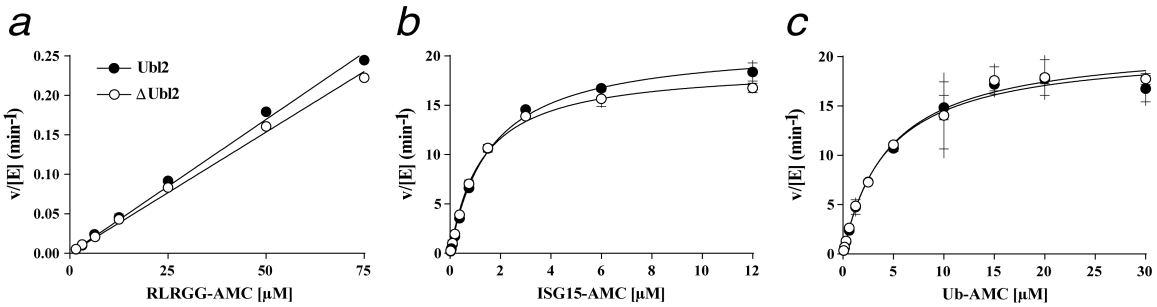

Supplementary Figure S2. *In vitro* analysis of F2124-0890 with proteases under non-reducing conditions reveals its lack of specificity and lack of potency under reducing conditions. Percent (%) inhibition was plotted as a function of increasing inhibitor concentrations in the absence of DTT (white circles) and presence of DTT (black circles) against viral and human proteases, MERS PLpro-Ubl2 (solid line, a) and PLpro-ΔUbl2 (dotted line, a), SARS PLpro (b), MHV PLP2 (c), USP7 (d), USP17 (e), and USP28 (f). Error bars represent the standard deviations obtained from triplicate data.

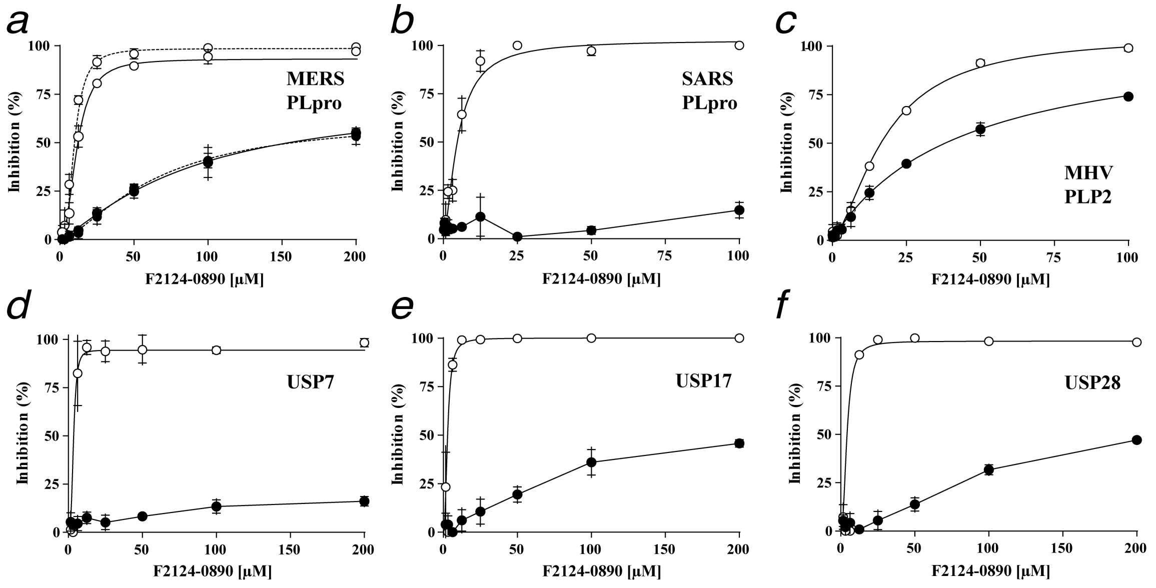

Supplement: Supplementary Information [file srep40292-s1.pdf]
